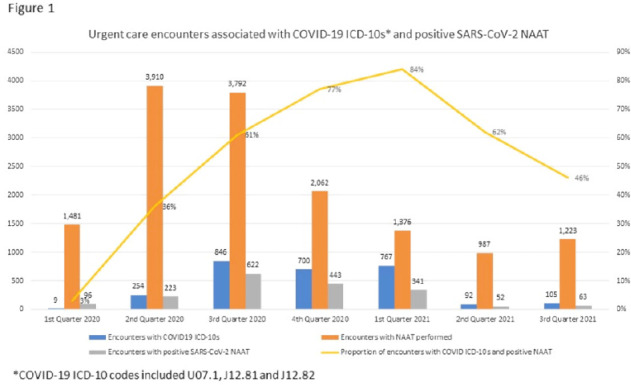# Impact of different COVID-19 encounter definitions on antibiotic prescribing rates in urgent care

**DOI:** 10.1017/ash.2022.63

**Published:** 2022-05-16

**Authors:** Sharon Onguti, David Ha, Emily Mui, Amy Chang, Eddie Stenehjem, Adam Hersh, Marisa Holubar

## Abstract

**Background:** Billing data have been used in the outpatient setting to identify targets for antimicrobial stewardship. However, COVID-19 ICD-10 codes are new, and the validity of using COVID-19 ICD-10 codes to accurately identify COVID-19 encounters is unknown. We investigated COVID-19 ICD-10 utilization in our urgent care clinics during the pandemic and the impact of using different COVID-19 encounter definitions on antibiotic prescribing rates (APRs). **Methods:** We included all telemedicine and office visits at 2 academic urgent-care clinics from January 2020 to September 2021. We extracted ICD-10 encounter codes and testing data from the electronic medical record. We compared encounters for which COVID-19 ICD-10 codes were present with encounters for which SARS-CoV-2 nucleic acid amplification testing (NAAT) was performed within 5 days of and up to 2 days after the encounter (Fig. 1). We calculated the sensitivity of the use of COVID-19 ICD-10 codes against a positive NAAT. We calculated the APR as the proportion of encounters in which an antibacterial drug was prescribed. This quality improvement project was deemed non–human-subjects research by the Stanford Panel on Human Subjects in Medical Research.

**Funding:** None

**Disclosures:** None

**Table:**
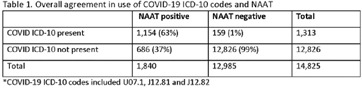

**Table:**
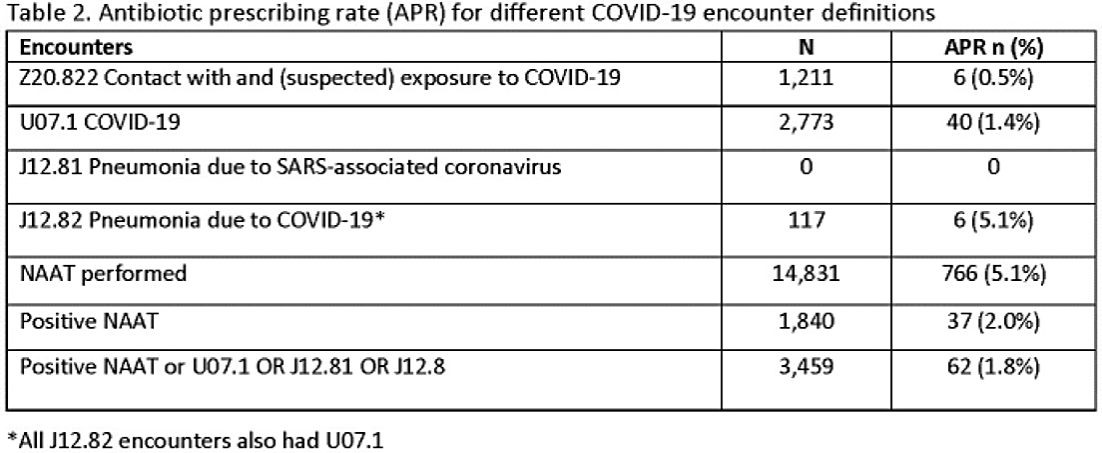

**Figure:**